# Mevalonate Kinase Deficiency and Squalene Synthase Inhibitor (TAK-475): The Balance to Extinguish the Inflammation

**DOI:** 10.3390/biom11101438

**Published:** 2021-09-30

**Authors:** Erika Rimondi, Erica Valencic, Alberto Tommasini, Paola Secchiero, Elisabetta Melloni, Annalisa Marcuzzi

**Affiliations:** 1 Department of Translational Medicine, University of Ferrara, 44121 Ferrara, Italy; erika.rimondi@unife.it (E.R.); paola.secchiero@unife.it (P.S.); elisabetta.melloni@unife.it (E.M.); annalisa.marcuzzi@unife.it (A.M.); 2 LTTA Centre, University of Ferrara, 44121 Ferrara, Italy; 3 Department of Paediatrics, Institute for Maternal and Child Health—IRCCS “Burlo Garofolo”, 34137 Trieste, Italy; erica.valencic@burlo.trieste.it; 4 Department of Medical Sciences, University of Trieste, 34127 Trieste, Italy

**Keywords:** mevalonate, inflammation, drug repositioning, rare disease

## Abstract

Mevalonate Kinase Deficiency (MKD) is a rare inborn disease belonging to the family of periodic fever syndromes. The MKD phenotype is characterized by systemic inflammation involving multiple organs, including the nervous system. Current anti-inflammatory approaches to MKD are only partially effective and do not act specifically on neural inflammation. According to the new emerging pharmacology trends, the repositioning of drugs from the indication for which they were originally intended to another one can make mechanistic-based medications easily available to treat rare diseases. According to this perspective, the squalene synthase inhibitor Lapaquistat (TAK-475), originally developed as a cholesterol-lowering drug, might find a new indication in MKD, by modulating the mevalonate cholesterol pathway, increasing the availability of anti-inflammatory isoprenoid intermediates. Using an in vitro model for MKD, we mimicked the blockade of the cholesterol pathway and evaluated the potential anti-inflammatory effect of Lapaquistat. The results obtained showed anti-inflammatory effects of Lapaquistat in association with a low blockade of the metabolic pathway, while this effect did not remain with a tighter blockade. On these bases, Lapaquistat could be configured as an effective treatment for MKD’s mild forms, in which the residual enzymatic activity is only reduced and not almost completely absent as in the severe forms.

## 1. Introduction

**Figure 1:**
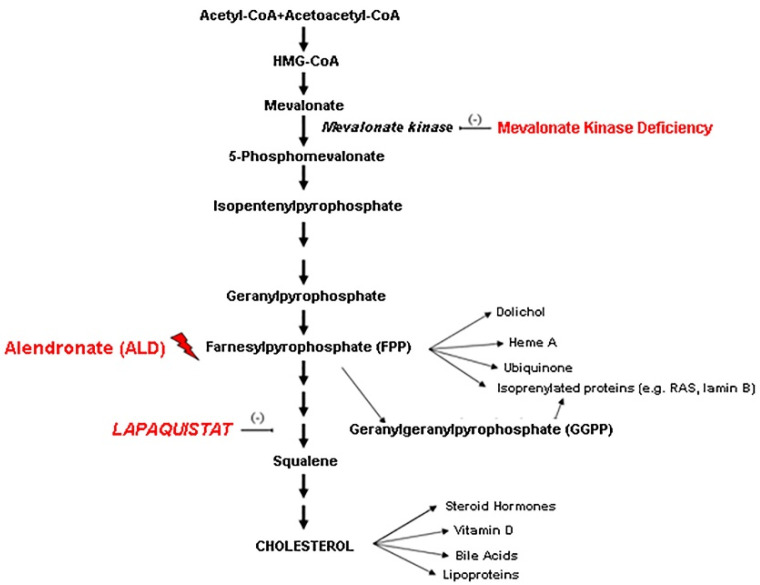
Schematic representation of the mevalonate pathway.

Mevalonate Kinase Deficiency (MKD) is a rare metabolic and autoinflammatory disorder caused by the mutation of the *MVK* gene (chromosome 12, q24). The mutation leads to a reduction in the mevalonate kinase enzyme activity, with a consequent decrease in a series of isoprenoid intermediate compounds and a mevalonate accumulation in plasma and urine in the acute phase [1] (Figure 1).

The clinical onset of the disease occurs in pediatric age but tends to last in life, presenting various degrees of severity, ranging from the less debilitating form (hyperimmunoglobulinemia D, HIDS, OMIM # 260920) to the most severe form (mevalonic aciduria, MA, OMIM # 610377). Both phenotypes are characterized by common symptoms that include periodic fever attacks associated with other inflammatory symptoms [2,3]. The common symptoms in HIDS are headache, splenomegaly, adenopathy, pharyngitis, abdominal and musculoskeletal pain. MA is also characterized, in addition to these symptoms, by significant psycho-motor and neurological involvement [4].

The most accredited hypothesis regarding MKD pathogenesis claims that the typical inflammation of the disease is caused by the deficiency of pre-squalenic isoprenoid intermediates, with a reduced prenylation of small GTPases, which, consequently, lose their membrane localization [5,6]. The lack of membrane-bound RhoA reflects on a reduced threshold of activation of the NLR family pyrin domain containing 3 (NLRP3) and pyrin inflammasomes and to the process of pyroptosis with a secretion of the inflammatory cytokines IL-1β, IL-6 and TNF-α [7,8,9]. An altered prenylation of KRAS (an isoform of RAS) is thought to play a role in the activation of PI3K-δ and in the lymphoproliferative phenotype characterizing MKD. Furthermore, the incorrect prenylation of the small GTPase Rab11 does not allow the formation of the autophagosome, leading to impaired mitophagy, which may contribute to the neurological damage observed in the most serious forms of MKD [10]. To date, MKD is no longer considered a treatment-orphan disease, because the IL-1 inhibitor treatments have allowed an acceptable control of the disease in most cases. However, not all patients fully respond to these therapies [11]. Furthermore, the anti-IL-1 biological treatments have a very high cost and require parenteral administration [12]. It remains, therefore, advisable to deepen the knowledge about the pathogenesis of MKD to identify more specific molecular targets for a mechanistic treatment.

Recent in vitro studies have shown a possible therapeutic role of a biochemical modulation of the mevalonate pathway [13]. For example, Lapaquistat (TAK-475), a compound belonging to the family of zaragozic acid, has been reported as a powerful competitive inhibitor of cholesterol synthesis in HepG2 cells [14]. Similarly, in a murine model, zaragozic acid was able to inhibit acute liver cholesterol synthesis accompanied by an accumulation of organic acids [15]. In summary, these experimental data highlighted the potential of squalene inhibitors in hypercholesterolemia [16]. The effect of zaragozic acid is related not only to the blockade of sterol synthesis, but also to a relative accumulation of geranylgeraniol due to both the blocking of its conversion to squalene and the induction of the mevalonate kinase [17]. Preliminary studies also suggest that this action could also help to protect neurons from a toxic effect that occurs under isoprenoid insufficiency conditions [18].

Of note, zaragozic acid could improve the MK residual enzymatic activity in skin fibroblasts derived from patients with MKD, increasing the transcription of the gene *Mvk* [13].

According to all evidence, TAK-475 could affect pathogenetic events relevant in MKD by increasing the levels of metabolites immediately upstream of the enzyme, including anti-inflammatory isoprenoids derived from the geraniol [19].

The study of the TAK-475 is also justified from the availability of safety data of the compound in phase three clinical trials at a lower dosage (50 mg/daily). However, the drug at a higher concentration (100 mg/daily) was halted because of a hepatic safety concern in addition to its comparable effectiveness as a cholesterol-lowering agent with respect to statins [20,21]. Nevertheless, even at a lower dosage, TAK-475 treatment led to reduction in C-reactive protein, highlighting its anti-inflammatory potential [22].

Therefore, the aim of the present study was to evaluate the potential of TAK-475 in our MKD model and, at the same time, to improve the understanding of the pathogenetic mechanism correlated to MKD.

## 2. Materials and Methods

### 2.1. Reagents

Lipopolysaccharide (LPS) (*E. coli*-serotype 055:B5) and alendronate (ALD), (Sigma-Aldrich, St. Louis, MO, USA) were dissolved in saline solution. Lapaquistat acetate (TAK-475, Shanghai iChemical Technology, Shanghai, China; purity 98%) was dissolved in ethanol before dilution in tissue culture medium, so that the final concentration of ethanol would not exceed 0.1% (*vol*/*vol*).

### 2.2. Cell Culture

RAW 264.7 cells (murine monocyte/macrophage cell line), obtained from Sigma-Aldrich, were cultured in Dulbecco’s modified eagle medium (DMEM) supplemented with 10% fetal bovine serum (both from EuroClone, Milan, Italy) and with L-glutamine and penicillin/streptomycin (GIBCO, Grand Island, NY, USA). Cells were left untreated or treated with 25 or 50 μM ALD for 20 h and, then, with 10 μg/mL LPS for an additional 24 h. TAK-475 (LAPA) was added together with ALD, when appropriate, at the concentration of 10 μM. At the end of the treatment time, the supernatants were collected for cytokines analyses, while cells were pelleted for the analysis of apoptosis, the electron microscopy studies and the Western blot assays.

### 2.3. Flow Cytometric Assessment of Apoptosis

The cytotoxic effects of ALD and LPS were assessed verifying apoptosis induction after Annexin V-FITC/propidium iodide (PI) double staining (Beckman Coulter Inc., Brea, CA, USA), according to the manufacturer’s instructions, using a FACSCalibur flow cytometer (BD Biosciences, San Josè, CA, USA) and the FlowJo software, version 9.9.6 (Tree Star, Ashland, OR, USA). The percentage of apoptotic cells correspond to the sum of A +/PI + (late apoptotic) and A +/PI − (early apoptotic) cells as shown in the Supplementary Figure S1.

### 2.4. The xCELLigence System and Impedance Measurement

The effect of the ALD, LPS and LAPA treatment on RAW 264.7 was evaluated by the xCELLigence RTCA DP Instrument (F. Hoffmann-La Roche SA, Basel, Switzerland), which records the increase in electrical impedance due to the presence of adherent cells on the well bottom, covered by microelectrodes. In real time and without the addition of a label, the number and the viability of attached cells are displayed as an alteration of the impedance that is converted in an adimensional parameter called “Cell Index” (CI). A decrease in CI after adding a pharmacological compound could be due to a detachment or to the death of cultured cells. Briefly, for this analysis, 5 × 10^3^ cells were seeded in a 16-well E-plate in 200 µL of complete medium, and cultured in 5% CO_2_ at 37 °C. The cells were stimulated with ALD, LPS and LAPA. The impedance was measured every 15 min; this experiment was reproduced thrice in triplicate. The change of impedance was calculated by dividing the values by the value of the untreated condition.

### 2.5. Measurement of Cytokines in Cell Culture Supernatants

Cytokine levels were measured in RAW 264.7 cell culture supernatant samples by performing a bead-based multiplex immunoassay (Bio-Plex assay, Bio-Rad Laboratories, Milan, Italy), including IL-6 and TNF-α, following the manufacturer’s instructions. Data were acquired using the Bio-Plex 200 reader, while a digital processor managed data output and Bio-Plex Manager^®^ 6.0 software presented data as Median Fluorescence Intensity and concentration (pg/mL) as well (Bio-Rad Laboratories).

### 2.6. Transmission Electron Microscopy

For Transmission Electron Microscopy, cells were treated as described above. Cells were then collected and centrifuged at 2000× *g* for 5 min and the pellets were fixed in 2.5% glutaraldehyde in 0.1M phosphate-buffered saline (PBS, pH 7.4) at 4 °C for 3 h. After several rinses with PBS, the cells were post-fixed in 2% buffered osmium tetroxide for 1 h. The samples were then dehydrated with graded concentrations of acetone and embedded in Araldite epoxy resin (Durcupan ACM, Fluka, Sigma-Aldrich Co., St. Louis, MO, USA) according to standard protocols. Semi-thin sections (1.5 μm) were cut on a Reichert Ultracut S ultramicrotome using glass knives, stained with a 1% toluidine blue solution and observed with an optical microscope (Nikon Eclipse E800). Ultrathin sections (90 nm) were prepared with an ultramicrotome (Reichert Ultracut S) and counterstained with uranyl acetate in a saturated solution and lead citrate according to Reynolds. The samples were observed under transmission electron microscope (Hitachi H800 at 100 Kv, Hitachi, Ltd., Tokyo, Japan).

### 2.7. Western Blot Analyses

RAW 264.7 cells, cultured and treated as reported above, were lysed in ice-cold RIPA buffer (50 mM Tris pH 7.5, 150 mM NaCl, 0.1% SDS, 1% Nonidet P-40, 0.25% sodium deoxycholate) supplemented with Pierce Protease and Phosphatase Inhibitor mini tablets (Thermo Scientific, Rockford, IL, USA) on ice for 1 h. Protein determination was performed by using the BCA Protein Assay (Thermo Scientific, Rockford, IL, USA). Samples were supplemented with the loading buffer (250 mM Tris pH 6.8, 2% SDS, 40% Glycerin, 20% b-mercaptoethanol) and boiled for 2 min. Equal amounts of protein for each sample were migrated in acrylamide gels and blotted onto nitrocellulose filters. Western blot analysis was performed according to standard procedures using the following primary antibodies: anti-LC3 and anti β-actin all from Sigma-Aldrich (St. Louis, MO, USA). After incubation with secondary antibodies (anti-mouse or -rabbit IgG HRP-conjugated; Sigma-Aldrich, St. Louis, MO, USA), a specific band detection was performed with the WesternBright Quantum kit (Advansta, Menlo Park, CA, USA). Images’ acquisition and analysis were performed using the ImageQuant™ LAS 4000 imager and TL software (GE Healthcare, Buckinghamshire, UK).

### 2.8. Data Analysis

All results were expressed as mean ± standard deviation. Statistical significance was calculated using two-way analysis of variance (ANOVA) and Bonferroni post-test. Analysis was performed through GraphPad Prism software, version 8.4.2 (GraphPad Software, La Jolla, CA, USA).

## 3. Results

### 3.1. Alendronate Plus LPS Induced Apoptosis on RAW 264.7 Cells

**Figure 2:**
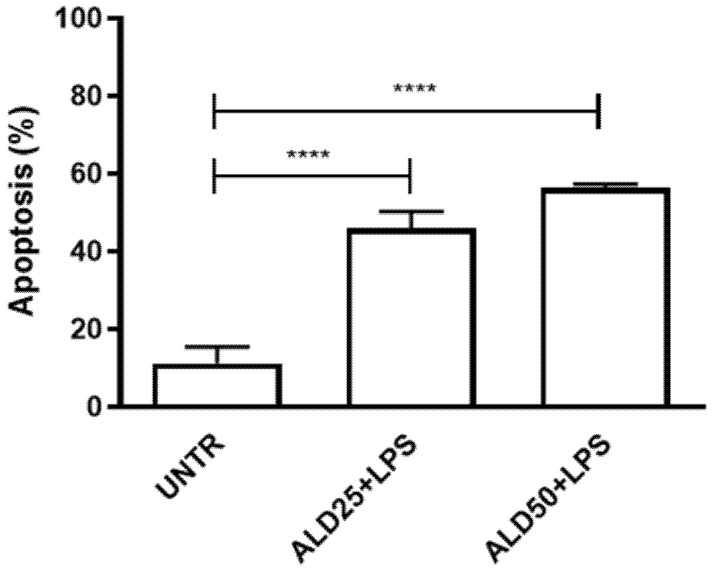
Analysis of RAW 264.7 apoptosis levels after treatment with two different ALD concentrations, 25 μM and 50 μM) plus LPS (10 μg/mL). Results are indicated as mean ± standard deviation of independent replicates, analyzed with ANOVA and corrected with Bonferroni post-test (**** *p* < 0.0001). UNTR: untreated.

The effect on cell viability of the combination of alendronate (ALD) and lipopolysaccharide (LPS), that mimics the MKD disease condition, was evaluated on RAW 264.7 cells by flow cytometry after treatment with 25 or 50 μM ALD for 48 h, in addition to 10 μg/mL LPS for 24 h. Results showed a significant induction of apoptosis after treatment with both 25 and 50 μM ALD, confirming a potent cytotoxic effect of this drug combination in our cellular model (Figure 2) (Untreated: 11.24 ± 4.24%; ALD25 + LPS: 45.95 ± 4.22%; ALD50 + LPS: 56.45 ± 0.83%; **** *p* < 0.0001). The single stimulus (ALD and LPS) did not induce a significant rate of apoptosis (ALD25: 21.38 ± 7.22%; ALD50: 24.50 ± 9.80%; LPS: 27.93 ± 7.09%) (data not shown).

### 3.2. Lapaquistat Counteracted the Cytotoxic Effect of Alendronate Plus LPS

The potential cytoprotective effect of Lapaquistat (LAPA) was evaluated on RAW 264.7 cells by the xCELLigence RTCA DP Instrument after 48 h of treatment with 10 μM of the drug in combination with 25 μM or 50 μM ALD and 24 h of treatment with 10 μg/mL LPS.

**Figure 3:**
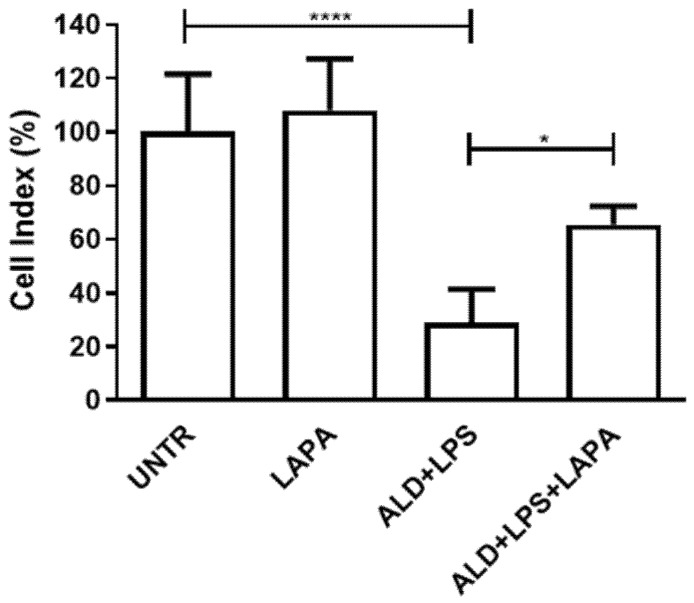
XCELLigence analysis of RAW 264.7 cell viability after treatment with ALD (50 μM), LPS (10 μg/mL) and 10 μM Lapaquistat (LAPA). Results are indicated as mean ± standard deviation of 3 independent replicates, analyzed with ANOVA and corrected with Bonferroni post-test (* *p* < 0.05, **** *p* < 0.0001). UNTR: untreated.

As shown in Figure 3, treatment with ALD (at the highest concentration, 50 μM) plus LPS significantly reduced the Cell Index with respect to untreated cells and this effect was clearly counteracted by Lapaquistat, that partially restored the Cell Index value (Untreated: 100.1 ± 21.33%; ALD + LPS: 28.86 ± 12.52%; LAPA + ALD + LPS: 65.50 ± 6.75%). The trend of ALD 25 μM was perfectly comparable to that of ALD 50 μM (data not shown). Of note, treatment with Lapaquistat alone did not show any cytotoxic effect in RAW 264.7 cells (LAPA: 108 ± 19.18%).

### 3.3. Anti-Inflammatory Effect of Lapaquistat in MKD Disease-Mimicking Conditions

**Figure 4:**
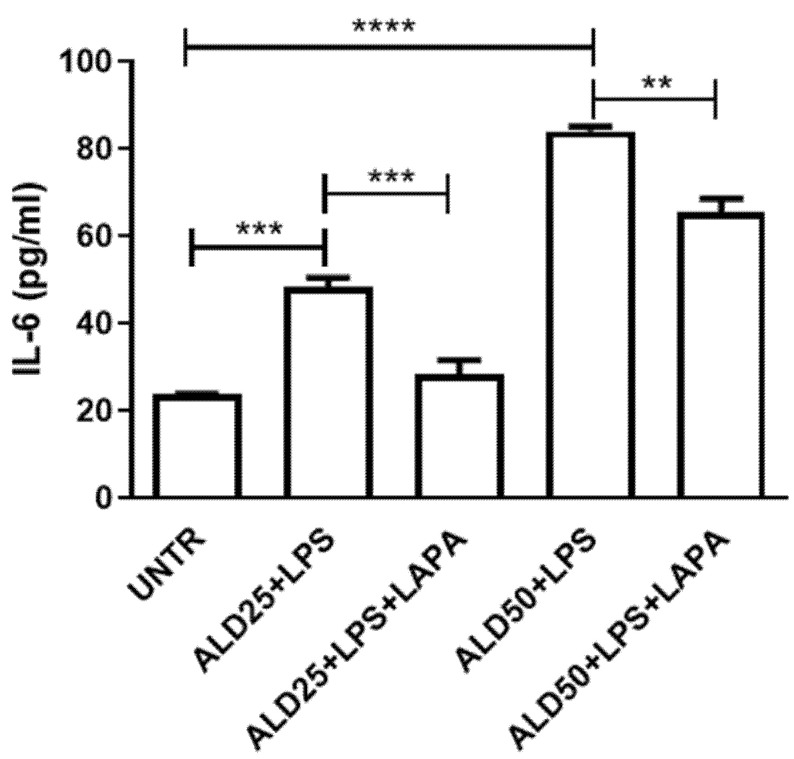
Analysis of IL-6 levels on supernatants of RAW 264.7 treated with ALD 25 μM or 50 μM plus 10 μg/mL LPS, with or without 10 μM Lapaquistat (LAPA). Data are expressed in pg/mL and indicated as mean ± SD of three independent experiments analyzed with ANOVA and corrected with Bonferroni post-test: ** *p* < 0.01, *** *p* < 0.001, **** *p* < 0.0001. UNTR: untreated.

**Figure 5:**
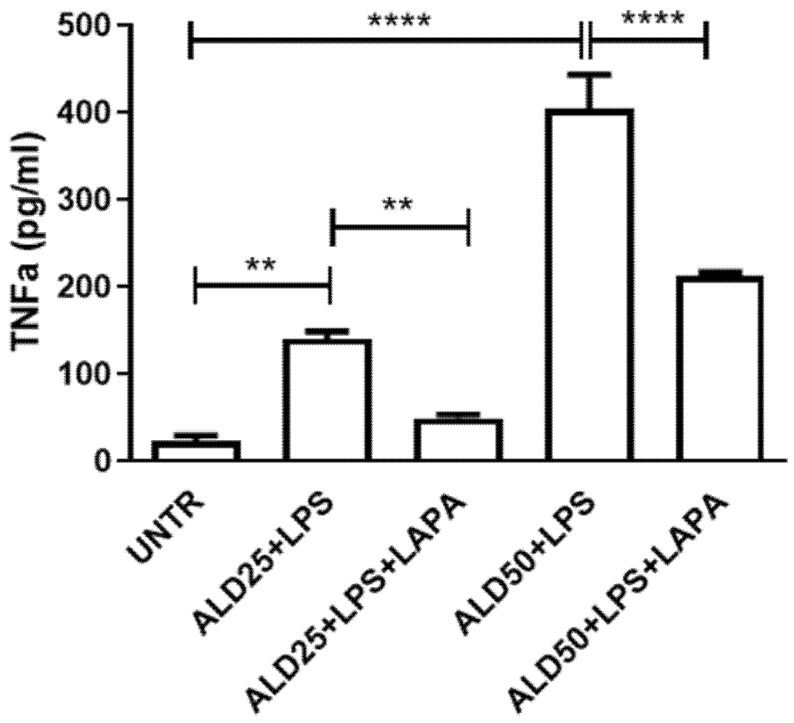
Analysis of TNF-α levels on supernatants of RAW 264.7 treated with ALD 25 μM or 50 μM plus 10 μg/mL LPS, with or without 10 μM Lapaquistat (LAPA). Data are expressed in pg/mL and indicated as mean ± SD of three independent experiments analyzed with ANOVA and corrected with Bonferroni post-test: ** *p* < 0.01, **** *p* < 0.0001. UNTR: untreated.

The levels of a wide panel of pro-inflammatory cytokines were firstly evaluated on RAW 264.7 cells treated for 48 h with 25 or 50 μM ALD and for the last 24 h with 10 μg/mL LPS. Among the cytokines analyzed, the treatment induced a significant increase in IL-6 (Figure 4) and TNF-α levels (Figure 5), but did not seem to affect the release of the other cytokines in the culture supernatants (data not shown). The simultaneous addition of Lapaquistat to ALD plus LPS treatment significantly reduced the induced levels of IL-6 and TNF-α (IL-6. Untreated: 23.72 ± 0.19; ALD25 + LPS: 48.39 ± 4.21; ALD25 + LPS + LAPA: 28.26 ± 6.66; ALD50 + LPS: 84.07 ± 2.03; ALD50 + LPS + LAPA: 65.44 ± 6.29) (TNF-α. Untreated: 23.43 ± 10.57; ALD25 + LPS: 140.6 ± 20.69; ALD25 + LPS + LAPA: 49.41 ± 8.92; ALD50 + LPS: 404.7 ± 78.19; ALD50 + LPS + LAPA: 212.9 ± 8.10). These data seemed to demonstrate the anti-inflammatory effect of Lapaquistat in our experimental MKD disease-mimicking setting.

### 3.4. Lapaquistat Counteracted the Mitochondrial Damage Induced by LPS and Alendronate

In order to investigate the protective role of Lapaquistat towards the cellular compartments involved in the mevalonate pathway, the morphological changes of RAW 264.7 cells were analyzed using a transmission electron microscope (TEM).

**Figure 6:**
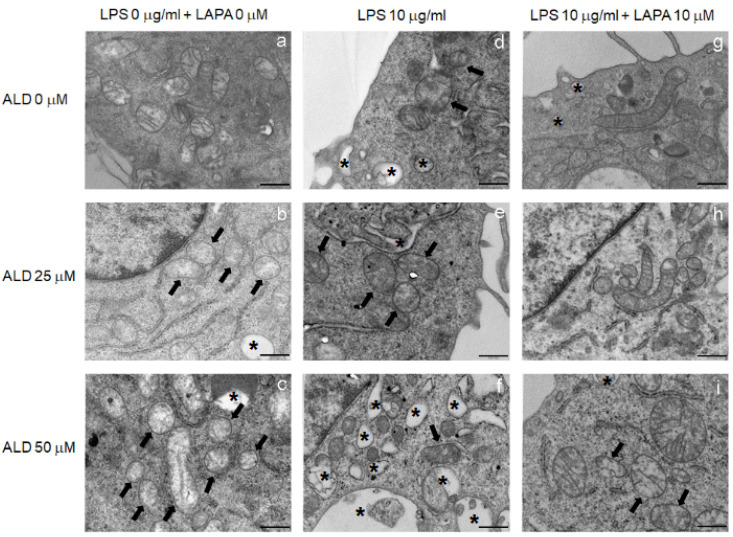
Morphological analysis of RAW 264.7 cells treated with ALD 25 μM or 50 μM plus 10 μg/mL LPS, with or without 10 μM Lapaquistat (LAPA). The ultrastructure of the cells was detected by transmission electron microscope (TEM). (**a**–**i**) Representative TEM images of RAW 264.7 cells treated as indicated in the figure labels, 25,000× magnification. Black arrows: swollen mitochondria. Asterisks: cytoplasmic vacuolations. Scale bars: 500 nm.

In the control condition (Figure 6a), cells showed a normal morphology, plasmatic and internal membranes were intact, cytoplasmic organelles and chromatin displayed a regular shape. As shown in Figure 6a, the mitochondria were round to oval in shape and presented well-defined cristae.

The addition of ALD 25 μM (Figure 6b) and ALD 50 μM (Figure 6c) for 48 h induced morphological modification, especially in mitochondria, directly related to the concentration of the bisphosphonate. A fraction of the mitochondria appeared modified with damaged cristae, mainly in the ALD 50 μM treatment. The treatment with LPS, added the last 24 h of incubation with ALD 25 μM (Figure 6e) and ALD 50 μM (Figure 6f), induced an extensive cytoplasmic vacuolation, more evident in ALD 50 μM + LPS, and the presence of swollen mitochondria with disrupted cristae. Cells were, then, treated with Lapaquistat alone or in combination with LPS and ALD 25 μM or ALD 50 μM. The morphological analyses showed that Lapaquistat was able to counteract the effects of LPS and ALD (Figure 6h,i) and that this activity was particularly evident in cells treated with a lower concentration of ALD (Figure 6h). RAW 264.7 treated with ALD 25 μM + LPS in combination with Lapaquistat displayed a reduction in the cytoplasmic vacuolation and most mitochondria with a normal morphology and well-defined cristae. The effect of Lapaquistat on ALD 50 μM + LPS treated cells was less evident, but cells still showed lower cellular damage and the presence of mitochondria with very clearly visible ridges, in addition to mitochondria that appeared empty with no cristae.

### 3.5. Lapaquistat Inhibits Autophagy in RAW 264.7 Cells

**Figure 7:**
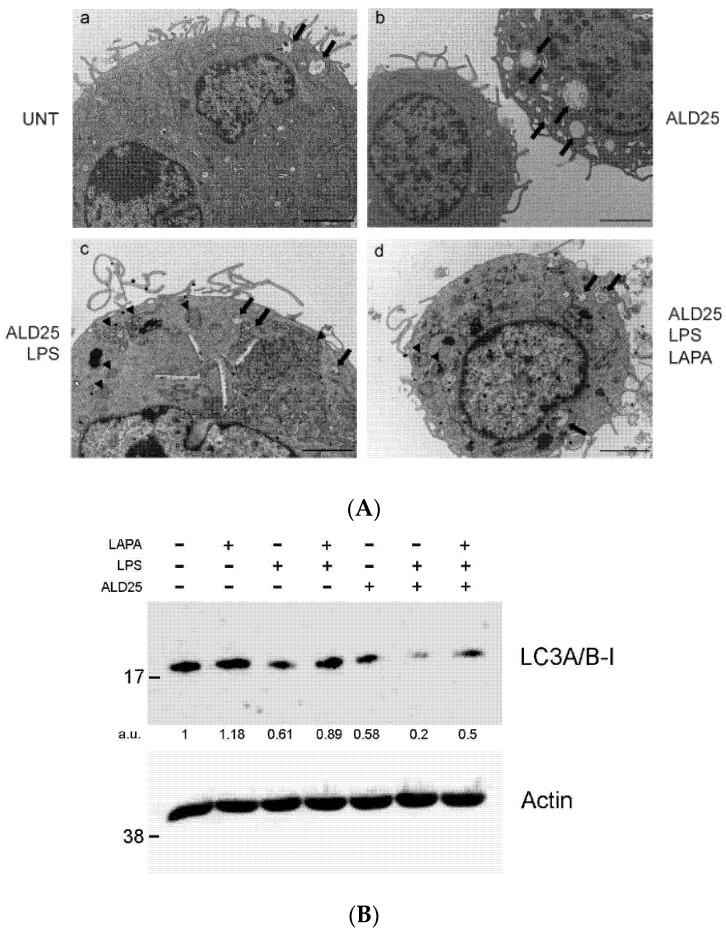
Evaluation of autophagy in RAW 264.7 treated with ALD 25 μM (ALD25) plus 10 μg/mL LPS, with or without 10 μM Lapaquistat (LAPA). (**A**) Representative TEM images of areas of cytoplasm, 8000× magnification. Scale bars: 2000 nm. Black arrowheads: mitophagosomes. Black arrows: autophagic compartments. (**B**) LC3 protein level was detected by Western blotting analysis. Three independent experiments were conducted; representative blot is shown. β-actin is shown as loading control. The ratio between LC3A-B I/β-actin densitometric quantification is indicated. a.u.: arbitrary unit.

Next, to investigate the mechanism of Lapaquistat, we further examined autophagy using TEM to visualize the presence of autophagosomes and autophagic compartments, based on their distinct ultrastructure [23]. As shown in Figure 7A (panel a), few autophagosomes were present in RAW 264.7 in the control condition. In samples treated with ALD 25 μM, we observed an increase in the number of cells presenting double membrane vesicles in the cytoplasm, indicative of autophagy (Figure 7A, panel b). The addition of LPS in combination with ALD 25 μM resulted in a strong increase in autophagosomes, with the presence of mitochondria surrounded by a double membrane typical of mitophagy [24], and autophagic compartments (Figure 7A, panel c). As shown in Figure 7A (panel d), the contemporary treatment with Lapaquistat reduced the presence of autophagosomes in the cytoplasm, suggesting the blockade of autophagy as a potential mechanism of action.

Starting from these evident morphological observations, we tried to analyze the autophagy-related protein LC3 by a Western blot analysis. As shown in Figure 7B, the treatment with LPS and ALD 25 μM induced a great decrease in LC3A/B-I protein expression and this decline was partially restored when Lapaquistat was added in combination with ALD 25 μM and LPS.

## 4. Discussion

MKD is an autosomal recessive rare disease, associated with mutations in the mevalonate kinase gene, coding for the homonymous enzyme that catalyzes the second step of the metabolic pathway that leads to the biosynthesis of cholesterol. The disease was discovered in 1980 and, to date, more than 250 mutations of this gene have been reported, all resulting in a significant decrease in residual enzyme activity (REA) [25]. The REA variability has allowed, over the years, to define a continuous spectrum of clinical phenotypes ranging from the less severe manifestation of the disease (HIDS) to the more severe one (MA). The pathogenic role of a decrement of mevalonate-derived intermediate compounds in MKD is well recognized: a GGPP-shortage is considered to have a key role in triggering the pathogenesis of the inflammatory MKD phenotype. Since 2016, MKD is no longer considered a treatment-orphan disease. Regulatory agencies have approved the extension of the therapeutic indications of the drug *Ilaris* (Canakinumab) previously prescribed for CAPS (periodic syndromes associated with cryopyrin), for active systemic juvenile idiopathic arthritis and for other diseases, including MKD, TRAPS (Tumor necrosis factor Receptor-Associated Periodic Syndrome) and FMF (Familial Mediterranean Fever) [11]. Previously, patients with MKD were treated with non-steroidal anti-inflammatory drugs, with poor effectiveness, with glucocorticoids, which were often burdened by serious adverse effects and by dependency, and with anti-cytokine biological drugs (anti TNF-α or anti IL-6) with partial benefit. The response to these pharmacological treatments in patients is variable and difficult to predict. This variability cannot be attributed to distinct MVK genotypes as patients with the same mutation may present significant differences in the clinical phenotype and response to medications. Among the hypotheses for these heterogeneous behaviors, epigenetic factors were suggested to have a discriminating role, but there is no doubt that further insights about MKD pathogenesis are necessary to give an answer to the many questions to establish a consolidated therapeutic line for MKD [26]. The study of TAK-475 can be inserted precisely in this context. The experimental design used in this study is a consolidated disease model that combines the inhibitory effect of alendronate to block the cholesterol pathway and LPS to mimic the inflammatory stimulus. As shown by the impedancemetry analysis performed in our study, TAK-475 preserved the viability of the experimental cell population treated with alendronate and LPS. In addition, a morphological observation by electron microscopy showed that the changes induced by alendronate could be prevented at least in part by the addition of Lapaquistat. Notably, the mitochondrial damage induced by alendronate and LPS treatment, characterized by the presence of swollen mitochondria in the cytoplasm, was almost completely counteracted by Lapaquistat, especially in cells treated with a lower concentration of alendronate. Moreover, a TEM observation evidenced the ability of Lapaquistat to reduce autophagy induced by alendronate and LPS. Autophagy is an active mechanism in the process of cellular stress and in the removal of damaged organelles and intracellular pathogens [27,28]. In our model, alendronate and LPS induced an increase in the amount of autophagosomes, frequently containing mitochondria and autophagic compartments, and this induction was counteracted by Lapaquistat. The effect of Lapaquistat on autophagy could be associated with its restoring action on the inflammatory response induced by alendronate and LPS.

Regarding the cytokine profile, as expected, the production of inflammatory cytokines IL-6 and TNF-α was significantly increased following treatment with LPS in cells treated with alendronate in a dose-dependent manner. At both doses of alendronate (25 and 50 μM), TAK-475 was able to reduce the release of these cytokines, decreasing the inflammatory condition. Transposing these results to MKD, we could hypothesize TAK-475 efficacy limited to the cases with a partial enzyme defect (as in the syndrome with Hyper-IgD), while it seems more unlikely that the drug could be useful for the more serious forms of mevalonic aciduria. Indeed, it is possible that with this drug, as previously reported with other enzymatic modulators, paradoxical effects may be obtained linked to the involvement of different pathogenetic mechanisms in MKD, depending on the severity of the defect.

From all this evidence, the data project us to continue the Lapaquistat studies to evaluate the possibility of its future application in the treatment of MKD and, perhaps, other inflammatory diseases, in view of the repositioning of the drug. It is known that other medications acting on the cholesterol pathway have controversial results when used in patients affected by MKD. For example, statins could lead to a mild improvement in some subjects with MKD [29], but severely worsened the crises in subjects with MA [30]. Alendronate has also been proposed for therapy on the basis of possible benefit in a single patient, but it may also worsen the deficiency in mevalonate-derived isoprenoids, leading to worse inflammation. Indeed, the cholesterol pathway is finely regulated in vivo, and it may be difficult to predict how in vitro studies may reflect on the development of clinical studies. Unfortunately, murine models closely reproducing the human disease are lacking, hindering the translation of new treatments to the clinics. Moreover, considering the fine regulation of the cholesterol pathway, we should also consider the possibility that the blockade of squalene synthase could worsen the lack of post-squalene sterols deficiency in MKD, with possible detrimental clinical consequences. Indeed, while the safety profile of Lapaquistat in healthy subjects is good at low doses, this is not warranted for subjects with MKD, in whom the blockade of squalene synthase may lead to an excessive suppression of the synthesis of sterol derived molecules. The administration of coenzyme Q10, vitamin D and E is already part of the support therapy in MA-patients and could be even more necessary [31] to reduce the potential adverse effects of the inhibition of squalene synthase, such as myopathy. Finally, the antiviral 25-hydroxycholesterol (25-HC) has also been found reduced in membranes of subjects with MKD [32] and could be more strongly reduced in the presence of squalene inhibitors.

Thus, more data are still necessary to propose a possible trial of Lapaquistat in subjects with MKD.

## 5. Conclusions

Repositioned drugs, such as Lapaquistat, can have the advantage of reducing development costs and times since pharmacokinetics, toxicology and safety data have been previously collected [33,34]. Moreover, although IL-1 inhibitors are showing positive results in clinical trials to control MKD inflammatory phenotypes, TAK-475 may have the theoretical advantage of acting more directly on the biochemical mechanisms involved in the disease over a wider spectrum of clinical manifestations and controlling inflammatory disorders. In addition, TAK-475 is an oral available medicine with a short half-life, and this could be a valuable benefit for patients.

TAK-475 acts, as described, downstream of the statins and allows the production of early intermediates of the cholesterol pathway (geranylgeranylation) that would otherwise be limited by the statins. According to this hypothesis, TAK-475 has already been tested in in vivo preclinical studies [20]. To date, no data on the effects of TAK-475 in MKD-patients are available, but some results regarding the effect of TAK-475 treatment in an in vitro model of MKD are available. Suzuki et al., indeed, recently demonstrated that TAK-475 significantly increases levels of isoprenoids derived from mevalonate (FPP, GGPP and farnesol), in a dose-dependent manner, in human monocytic cells THP-1 [35].

The study of drugs that act on the mevalonate pathway, in general, has allowed us to control the mechanisms that go beyond the production of cholesterol. These results support the hypothesis about a potential pathogenic contiguity between different autoinflammatory diseases; therefore, it is possible that treatment with TAK-475 may also be useful for other autoinflammatory syndromes.

## Data Availability

Not applicable.

## Supplementary Materials

The following are available online at https://www.mdpi.com/article/10.3390/biom11101438/s1, Figure S1: exemplificative figure showing FACS analysis of apoptosis.
