# Supplementary material for: Mevalonate Kinase Deficiency and Squalene Synthase Inhibitor (TAK-475): The Balance to Extinguish the Inflammation
Source: Biomolecules. 2021 Sep 30;11(10):1438. doi: 10.3390/biom11101438 (PMC8533390; doi:10.3390/biom11101438)
Supplement: Supplementary file 1 [file biomolecules-11-01438-s001.zip › biomolecules-1379540-supplementary.pdf]

Supplementary Material

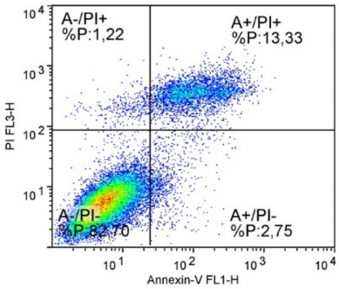

Supplementary Figure 1. Exemplificative figure showing FACS analysis of apoptosis, after double staining with Annexin V and Propidium Iodide. The apoptotic cells correspond to the sum of A+/PI+ (late apoptotic) and A+/PI- (early apoptotic) cells.
